# Supplementary material for: PacBio long-read amplicon sequencing enables scalable high-resolution population allele typing of the complex CYP2D6 locus
Source: Commun Biol. 2022 Feb 25;5:168. doi: 10.1038/s42003-022-03102-8 (PMC8881578; doi:10.1038/s42003-022-03102-8)
Supplement: Supplementary file 2 — Supplementary Information [file 42003_2022_3102_MOESM2_ESM.pdf]

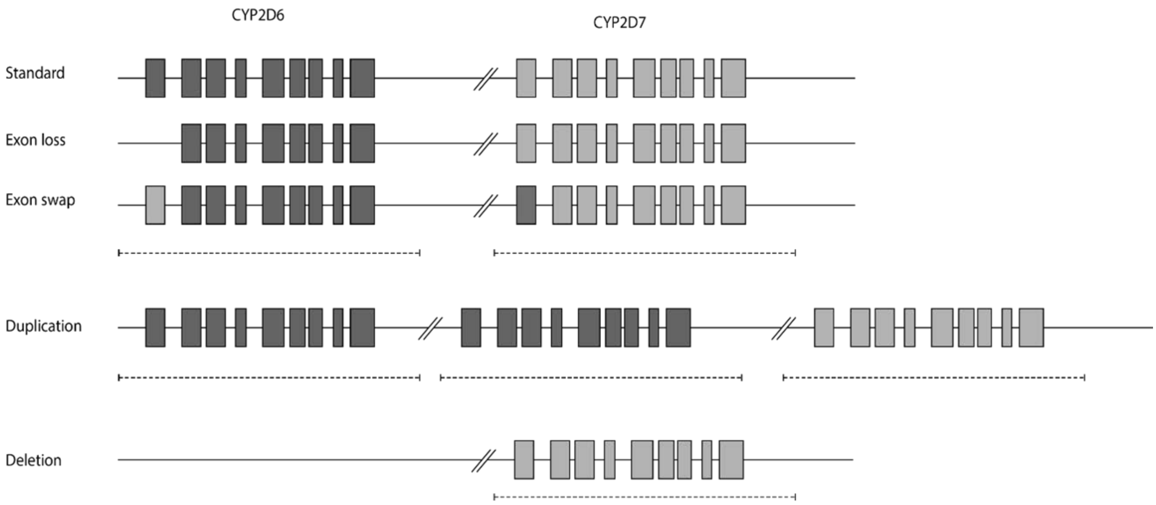

Supplementary Figure 1: CYP2D6 structural variants and long-amplicon PCR.

CYP2D6 (dark grey) has multiple variants. As well as SNPs which can cause reduced or loss of function, a variety of structural variants have been identified. These include allele conversions with *CYP2D7* (light grey) which may cause loss of function (e.g., *4.013 and *35.002), hybrids with *CYP2D7* often causing loss of function (e.g., *10.003 and *13), multiple gene duplications (*xN alleles) and gene deletion (*5). The PCR was developed to amplify *CYP2D6* and *CYP2D7* in each of these situations (dotted line). It will not amplify the region in which *CYP2D6* is deleted, in which instance the presence of *CYP2D7* will act as a control for DNA quality and PCR. In *1 ('normal') the amplicons will be 6.1kb for *CYP2D6* and 7.6kb for *CYP2D7*. The size will vary depending on SVs.

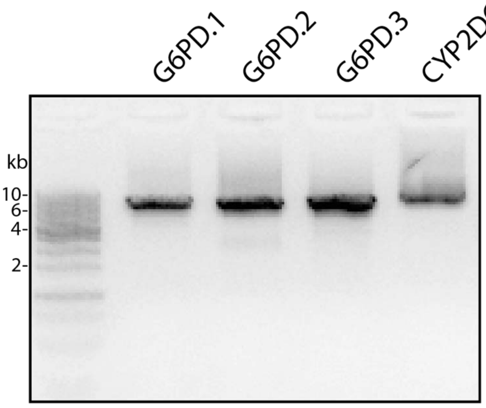

Supplementary Figure 2: Gene specific PCR products.

Gene specific primer pairs were designed and appended to overhangs. PCR gel shows specific bands for *G6PD* (in 3 parts, not part of this manuscript) and *CYP2D6* using mixed human DNA (Promega).

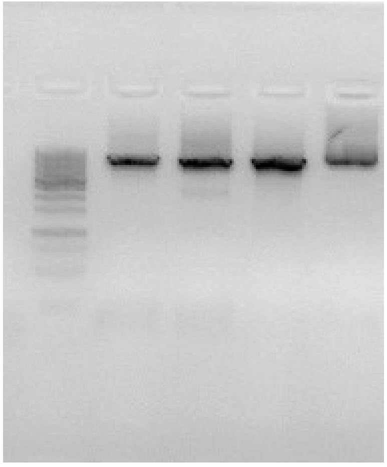

Supplementary Figure 3: Uncropped Gel for Supplementary Figure 2.

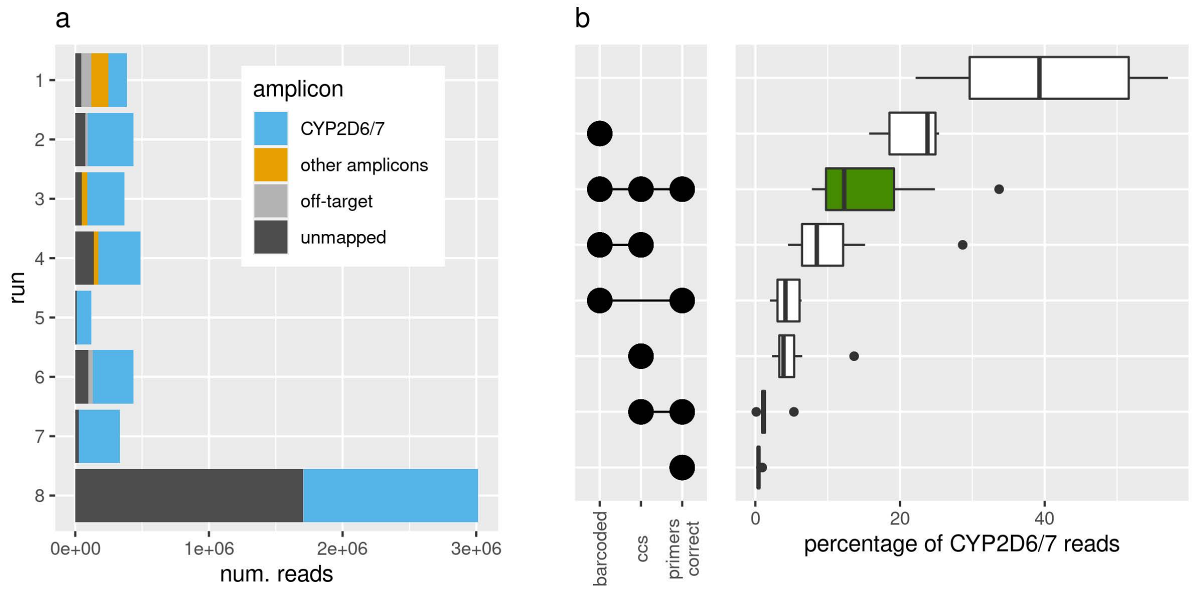

Supplementary Figure 4: SMRT run summary plots

a Number and status of polymerase reads in each run. Note that run 8 was performed on the Sequel II platform which accounts for the greater number of reads; **b** Tukey boxplots showing the distribution of reads assigned to *CYP2D6/D7* across sequencing runs (n = 8) in each filter state. The y-axis shows a truth-table with circles indicating the filter state of the reads (e.g., the top row show reads that were not able to be barcoded (demultiplexed), not able to form CCS and without correct sequencing primers). The filled boxplot shows the distribution of reads which passed all three filters.

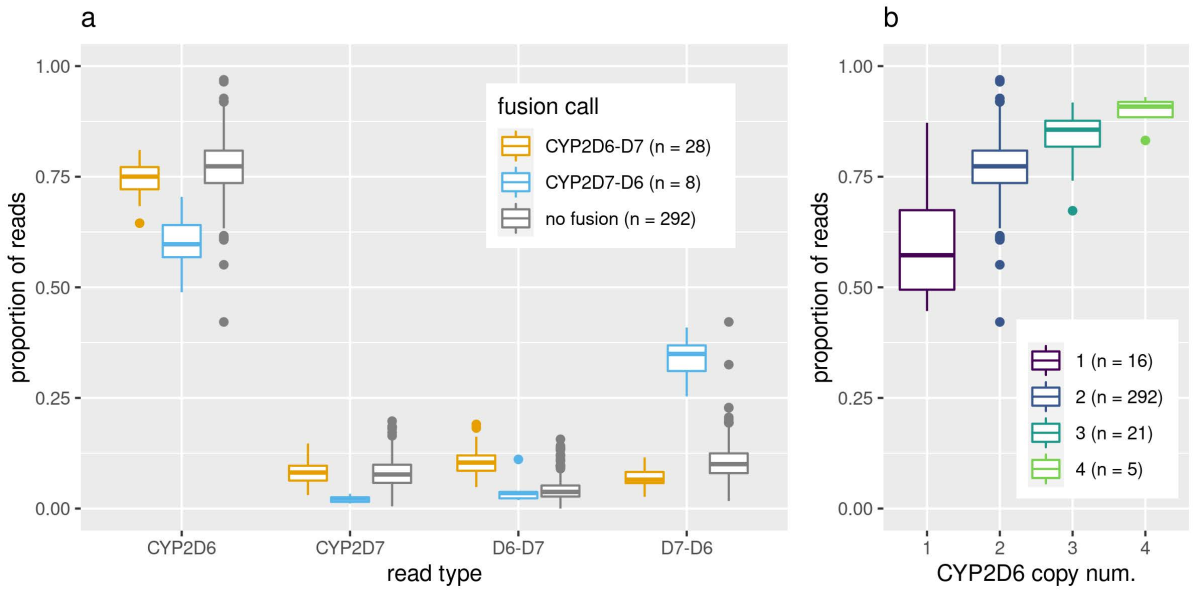

Supplementary Figure 5: *CYP2D6*, *CYP2D7* and fusion/chimeric read proportions

a Tukey boxplots showing the proportion of reads assigned to *CYP2D6*, *CYP2D7* and D6-D7/D7-D6 chimera/fusion by fusion call across samples. Note that as expected fewer reads are assigned to CYP2D6 in the presence of a fusion, and conversely more reads are assigned to the corresponding fusion. Shown only are samples with at least 50 reads and *CYP2D6* copy number of 2 (n=328); **b** Tukey boxplots showing the proportion of *CYP2D6* reads across samples versus *CYP2D6* copy number. The proportion of reads is observed to increase with increasing *CYP2D6* copy number. Shown only are samples with at least 50 reads and no fusion alleles called (n=334).

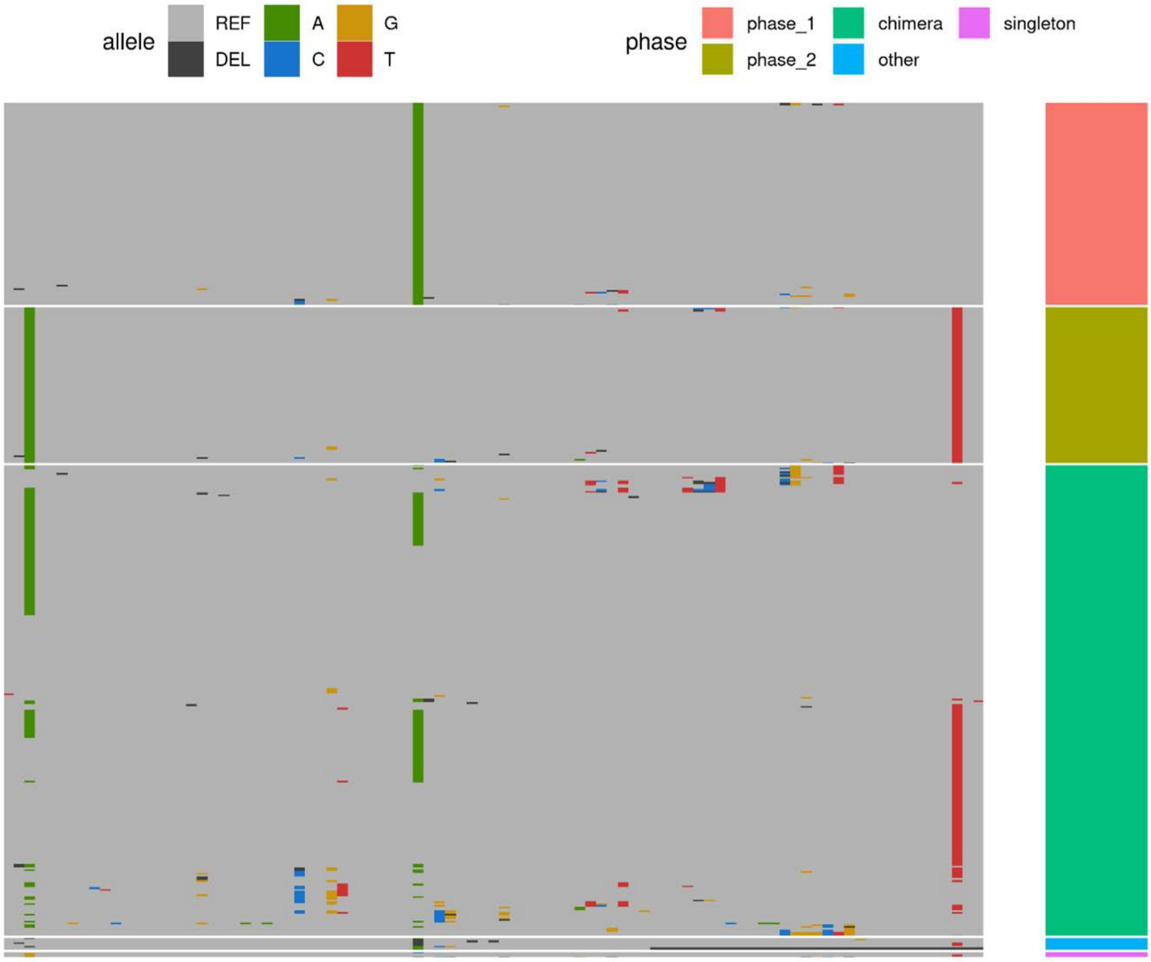

Supplementary Figure 6: AmpPhaseR *CYP2D6* Read phasing visualization for sample NA17052

The tabular visualization generated by the AmpPhaseR package represents individual CCS reads in rows and polymorphic SNP positions in columns, wherein each cell is colored by the allele present. In this case multiple chimeric species are observed derived from the two identified phases (alleles).

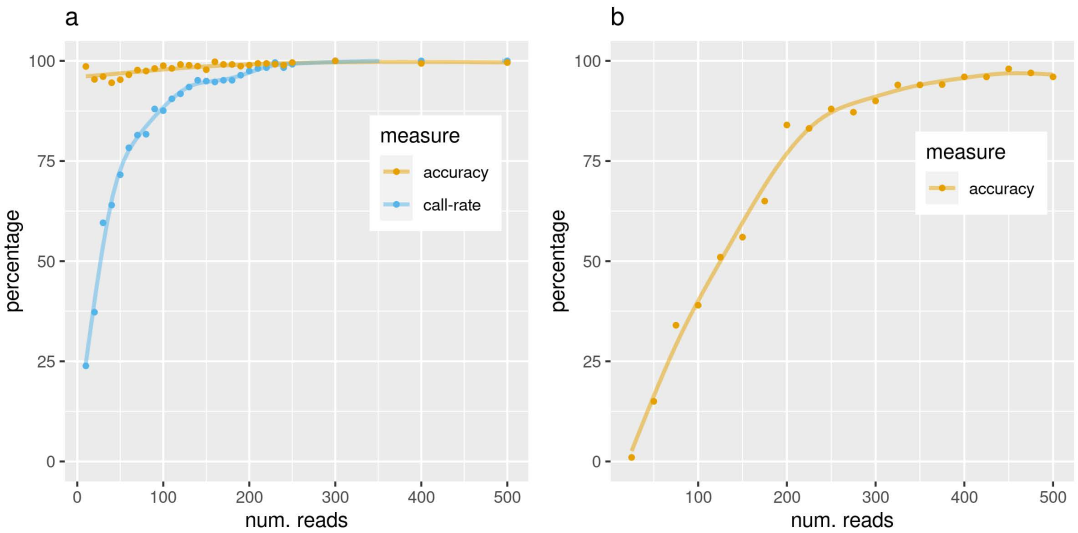

Supplementary Figure 7: Downsampling analysis

a Effect of downsampling *CYP2D6* CCS reads on call-rate and accuracy across 47 samples; **b** Effect of downsampling *CYP2D6*/*CYP2D7* CCS reads on accuracy of fusion calls across 20 samples. Smoothed trend lines are generated using a generalized additive model (GAM). Accuracy and call-rate measures at each point are averaged over five independent replicate runs.

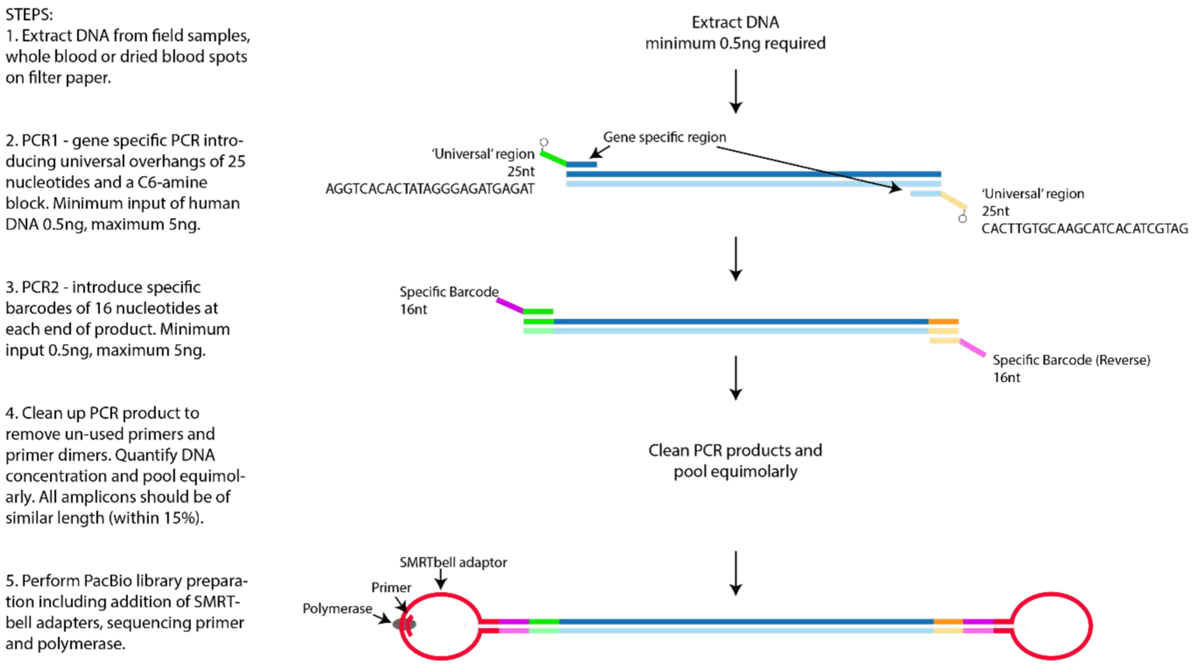

Supplementary Figure 8: Sample preparation flowchart.

Samples extracted from field samples with minimal DNA can be sequenced. Gene specific primer pairs are designed with an overhang sequence and have a C6-amine block. The same set of 96 barcoded primers can then be used with any gene specific primer pair. Products are cleaned and pooled prior to entering the library preparation process.
