# Supplementary material for: PacBio long-read amplicon sequencing enables scalable high-resolution population allele typing of the complex CYP2D6 locus
Source: Commun Biol. 2022 Feb 25;5:168. doi: 10.1038/s42003-022-03102-8 (PMC8881578; doi:10.1038/s42003-022-03102-8)
Supplement: Supplementary file 3 — Description of Additional Supplementary Files [file 42003_2022_3102_MOESM3_ESM.pdf]

Description of Additional Supplementary Files

File name: Supplementary Data 1-6
Description:

Supplementary Data 1: Sample storage effect on long amplicon PCR success. Samples were either extracted from finger prick blood collected directly into an EDTA tube (approximately 200 µL blood) and stored at -20˚ C, or extracted onto blotting paper (Dried blood spot, DBS, approximately 50 µL blood) and stored at room temperature prior to extraction. After extraction all samples were stored in a controlled -20˚ C environment. Percentage CYP2D6 PCR positive (and number positive of total tested, n) assessed by a positive band on an ethidium bromide stained gel, or by an amplification of at least 10 fold by PicoGreen assay. Sample sources are Victorian Blood Donor Registry (VBDR) in Melbourne Australia, samples from a clinical study in Solomon Islands, and samples from a study in Lihir, Papua New Guinea.

Supplementary Data 2: Primer sequences used for long amplicon PCR of gene specific regions

Supplementary Data 3: SMRT run summary statistics. Summary statistics from each of the SMRT sequencing runs conducted for this study. "Num. samples" indicates the total number of samples multiplexed in each run, with the amount in brackets indicating the number the number specific to the CYP2D6/7 amplicon. "Read Type" was assigned based on mapping the read against the GRCh38 reference genome.

Supplementary Data 4: Sample copy number and allele typing results. Columns "Sample", "Cohort", "Intron-2 copy num.", "Exon-9 copy num." and "Fusion allele" contain duplicated information in the case a sample contains multiple distinct alleles. The column "Allele copy num" indicates the number of copies of an allele, so homozygous samples will only occupy a single row in the table. See Supplementary Data 5 for allele definitions, and Supplementary Data 6 for sample activity scores and metaboliser status.

Supplementary Data 5: CYP2D6 allele definitions. Variants are given in the form "chromosome-position-reference_allele-alternate_allele" and are based on reference GRCh38. Novel "Star alleles" N1-N7 are not part of the PharmVar nomenclature and have not yet been assigned Star Allele IDs. These are assigned based on pressence of variants with a VEP predicted impact of MODERATE or HIGH. Simlarly novel suballeles of the form "*XX.novel-XX" do not match any described suballele in PharmVar.

Supplementary Data 6: Sample metaboliser status. Sample activity scores calculated from the “CYP2D6 Allele Functionality Table” (PharmGKB).

File name: Supplementary Data 7
Description: Source data for figures.
