# Supplementary material for: PacBio long-read amplicon sequencing enables scalable high-resolution population allele typing of the complex CYP2D6 locus
Source: Commun Biol. 2022 Feb 25;5:168. doi: 10.1038/s42003-022-03102-8 (PMC8881578; doi:10.1038/s42003-022-03102-8)
Supplement: Supplementary file 6 — Reporting Summary [file 42003_2022_3102_MOESM6_ESM.pdf]

nature portfolio

Corresponding author(s): Prof Melanie Bahlo

Last updated by author(s): Jan 12 2022

Reporting Summary

Nature Portfolio wishes to improve the reproducibility of the work that we publish. This form provides structure for consistency and transparency in reporting. For further information on Nature Portfolio policies, see our Editorial Policies and the Editorial Policy Checklist.

Statistics

For all statistical analyses, confirm that the following items are present in the figure legend, table legend, main text, or Methods section.

n/a	Confirmed	
☐	☒	The exact sample size (n) for each experimental group/condition, given as a discrete number and unit of measurement
☒	☐	A statement on whether measurements were taken from distinct samples or whether the same sample was measured repeatedly
☐	☒	The statistical test(s) used AND whether they are one- or two-sided<br>*Only common tests should be described solely by name; describe more complex techniques in the Methods section.*
☒	☐	A description of all covariates tested
☒	☐	A description of any assumptions or corrections, such as tests of normality and adjustment for multiple comparisons
☐	☒	A full description of the statistical parameters including central tendency (e.g. means) or other basic estimates (e.g. regression coefficient) AND variation (e.g. standard deviation) or associated estimates of uncertainty (e.g. confidence intervals)
☐	☒	For null hypothesis testing, the test statistic (e.g. F, t, r) with confidence intervals, effect sizes, degrees of freedom and P value noted<br>*Give P values as exact values whenever suitable.*
☒	☐	For Bayesian analysis, information on the choice of priors and Markov chain Monte Carlo settings
☒	☐	For hierarchical and complex designs, identification of the appropriate level for tests and full reporting of outcomes
☒	☐	Estimates of effect sizes (e.g. Cohen's d, Pearson's r), indicating how they were calculated

Our web collection on statistics for biologists contains articles on many of the points above.

Software and code

Policy information about availability of computer code

Data collection	LightCycler 480 software (version 1.5.1.62) for qPCR data.<br>PacBio Sequel CtrlVer 6.0.1.52258 and SigProcVer 6.0.0.47712 for PacBio SMRT subreads.bam files.
Data analysis	Software used for the PLASTER pipeline is available in a docker container hosted on docker hub at bahlolab/plaster:v0.0, from which individual software versions can be obtained.<br><br>For the qPCR data analysis, the following software was used: R version 3.6.1 with packages dplyr version 1.0.2, ggplot2 version 3.3.2, magrittr version 1.5, readr version 1.3.1, tidyr version and mixtools version 1.2.<br><br>Custom software used is available at https://github.com/bahlolab/PLASTER.

For manuscripts utilizing custom algorithms or software that are central to the research but not yet described in published literature, software must be made available to editors and reviewers. We strongly encourage code deposition in a community repository (e.g. GitHub). See the Nature Portfolio guidelines for submitting code & software for further information.

Data

Policy information about availability of data

All manuscripts must include a data availability statement. This statement should provide the following information, where applicable:

- Accession codes, unique identifiers, or web links for publicly available datasets
- A description of any restrictions on data availability
- For clinical datasets or third party data, please ensure that the statement adheres to our policy

The PacBio CCS data (output from the PLASTER pre-processing stage) is available through the NCBI Sequence Read Archive (SRA) (BioProject Accession PRJNA754842). Raw qPCR data is available in the PLASTER GitHub repository (https://github.com/bahlolab/PLASTER).

Field-specific reporting

Please select the one below that is the best fit for your research. If you are not sure, read the appropriate sections before making your selection.

☒ Life sciences ☐ Behavioural & social sciences ☐ Ecological, evolutionary & environmental sciences

For a reference copy of the document with all sections, see nature.com/documents/nr-reporting-summary-flat.pdf

Life sciences study design

All studies must disclose on these points even when the disclosure is negative.

Sample size	The Solomon Islands Cohort was gathered as part of clinical trial that is not discussed or reported on in this manuscript. The sample size was determined based on the requirements of the clinical trial.
Data exclusions	Samples were excluded when insufficient sequencing depth was attained for allele-typing.
Replication	10 Control samples with previously characterized CYP2D6 alleles from the Coriell Biobank were included, and concordance of results was assessed.
Randomization	N/A
Blinding	N/A

Reporting for specific materials, systems and methods

We require information from authors about some types of materials, experimental systems and methods used in many studies. Here, indicate whether each material, system or method listed is relevant to your study. If you are not sure if a list item applies to your research, read the appropriate section before selecting a response.

Materials & experimental systems

n/a	Involved in the study	
☒	☐	Antibodies
☒	☐	Eukaryotic cell lines
☒	☐	Palaeontology and archaeology
☒	☐	Animals and other organisms
☐	☒	Human research participants
☒	☐	Clinical data
☒	☐	Dual use research of concern

Methods

n/a	Involved in the study	
☒	☐	ChIP-seq
☒	☐	Flow cytometry
☒	☐	MRI-based neuroimaging

Human research participants

Policy information about studies involving human research participants

Population characteristics	Population from the Tetere region of the Solomon Islands.
Recruitment	Individuals were recruited through local health care centers, inclusion criteria was positive diagnosis of vivax malaria. Exclusion criteria were infancy (< 1 year), pregnancy, a positive G6PD deficiency test or refusal or inability to provide informed written consent. The inclusion/exclusion criteria are unlikely to bias the results of the CYP2D6 allelic characterisation of this population.

Ethics oversight	This study was approved by the Solomon Islands Health Research Ethics Review Board (HRE #041/16) and the Walter and Eliza Hall Institute Human Research Ethics Committee (HREC #16/02).

Note that full information on the approval of the study protocol must also be provided in the manuscript.
